# Evidence for reticulospinal plasticity underlying motor recovery in Brown-Séquard-plus Syndrome: a case report

**DOI:** 10.3389/fneur.2024.1335795

**Published:** 2024-06-04

**Authors:** Antonia Maria Eilfort, Maria Rasenack, Björn Zörner, Armin Curt, Linard Filli

**Affiliations:** ^1^Spinal Cord Injury Center, Balgrist University Hospital, Zurich, Switzerland; ^2^Neuroscience Center Zurich, University of Zurich, Zurich, Switzerland; ^3^Department of Health Science and Technology, ETH Zurich, Zurich, Switzerland; ^4^Swiss Paraplegic Center and Swiss Paraplegic Research, Nottwil, Switzerland; ^5^Swiss Center for Movement Analysis, Balgrist Campus AG, Zurich, Switzerland

**Keywords:** Brown-Séquard Syndrome, spinal cord injury, reticulospinal tract, corticospinal tract, StartReact, motor recovery, neural plasticity, mirror activity

## Abstract

Brown-Séquard Syndrome (BSS) is a rare neurological condition caused by a unilateral spinal cord injury (SCI). Upon initial ipsilesional hemiplegia, patients with BSS typically show substantial functional recovery over time. Preclinical studies on experimental BSS demonstrated that spontaneous neuroplasticity in descending motor systems is a key mechanism promoting functional recovery. The reticulospinal (RS) system is one of the main descending motor systems showing a remarkably high ability for neuroplastic adaptations after incomplete SCI. In humans, little is known about the contribution of RS plasticity to functional restoration after SCI. Here, we investigated RS motor drive to different muscles in a subject with Brown-Séquard-plus Syndrome (BSPS) five months post-injury using the StartReact paradigm. RS drive was compared between ipsi- and contralesional muscles, and associated with measures of functional recovery. Additionally, corticospinal (CS) drive was investigated using transcranial magnetic stimulation (TMS) in a subset of muscles. The biceps brachii showed a substantial enhancement of RS drive on the ipsi- vs. contralesional side, whereas no signs of CS plasticity were found ipsilesionally. This finding implies that motor recovery of ipsilesional elbow flexion is primarily driven by the RS system. Results were inversed for the ipsilesional tibialis anterior, where RS drive was not augmented, but motor-evoked potentials recovered over six months post-injury, suggesting that CS plasticity contributed to improvements in ankle dorsiflexion. Our findings indicate that the role of RS and CS plasticity in motor recovery differs between muscles, with CS plasticity being essential for the restoration of distal extremity motor function, and RS plasticity being important for the functional recovery of proximal flexor muscles after SCI in humans.

## 1 Background

Brown-Séquard Syndrome (BSS) and Brown-Séquard-plus Syndrome (BSPS) are uncommon conditions occurring in 2–4% of patients with traumatic spinal cord injury (SCI) (1, 2). A pure form of BSS is extremely rare and characterized by a confined unilateral spinal lesion that results in ipsilesional hemiplegia and loss of proprioception, as well as loss of pain and temperature sensation on the contralesional side below the level of injury (3). Most observations of Brown-Séquard-like syndromes correspond to the less pure form of the syndrome termed BSPS. The BSPS consists of a predominantly unilateral spinal lesion that leads to a characteristic asymmetric presentation of paresis and hypalgesia (2, 3). Patients with BSS and BSPS generally have a good prognosis for motor recovery and for a return to near pre-injury lifestyles (3, 4).

An important driver for functional recovery after incomplete SCI is spontaneous neuroplastic adaptation within the central nervous system (CNS) (5). The predominant impairment of descending fibers on one side, and sparing of fibers on the other, renders the BSS an interesting model to study neuroplasticity and functional recovery. Fiber sprouting and synaptic rewiring of descending motor tract systems lead to enhanced motor drive in spinal cord areas below the lesion, inducing functional recovery. This has been extensively demonstrated for the corticospinal (CS) system (4, 6). However, neuroplasticity has also been shown in other descending motor systems such as the phylogenetically conserved, functionally relevant reticulospinal (RS) system. Besides the CS system, the RS system is considered a main descending motor system for movement control (7). Recent preclinical studies revealed that the RS system shows a high potential for neuroplastic adaptations upon incomplete SCI: Preserved RS fibers showed remarkable compensatory sprouting, with axons crossing the midline of the sublesional spinal cord, innervating the ipsilesional (i.e., denervated) hemicord (8, 9). Additionally, other preclinical BSS studies identified significant regenerative sprouting of severed RS fibers above the level of injury forming new synapses onto propriospinal neurons bypassing the lesion site (10). Recently, Asboth and colleagues found that, after a severe experimental SCI transecting the CS tract in mice, fibers from the motor cortex synapsed onto sprouting descending RS fibers, thereby forming a cortico-reticulospinal detour pathway (11). The mentioned studies indicate that RS plasticity is a key mechanism driving functional recovery in experimental SCI models. There is growing evidence that RS plasticity might also play an essential role in the restoration of motor function in humans with SCI (12, 13).

The most common approach to assess RS drive non-invasively is the StartReact paradigm (14). In this paradigm, the reaction time of movements is shortened when movement initiation is paired with loud acoustic stimuli (LAS) compared to moderate acoustic stimuli (MAS). Although the mechanisms underlying the StartReact effect are not fully understood, there is compelling evidence from preclinical (15) and clinical studies (13, 16) that the RS system plays a key role in the reaction time shortening. Indeed, the degree of reaction time shortening is hypothesized to reflect the extent of the RS drive (12, 15, 17). The StartReact paradigm has previously been used to assess RS plasticity in neurological patients (13, 16).

Compared to the CS system, RS projections are more diffuse, often innervating spinal interneurons and motor neurons of both ipsi- and contralateral sides (18). Previous studies have demonstrated that single RS neurons drive bilateral muscle activation (19–21). Upregulation of the RS system has been associated with mirror movements, describing involuntary synchronous movements of one limb during voluntary movements of the other limb (22, 23). Mirror movements are often reported in stroke patients, and are usually observed in the less impaired extremity, during voluntary activation of the more impaired extremity (23, 24). Mirror movements quantified by electromyographic (EMG) recordings are typically referred to as mirror activity.

This study aimed at examining neuroplasticity in the two principal desending motor systems, i.e., the CS and RS system, in a patient with BSPS. We hypothesized that RS plasticity is present in proximal muscles after SCI, resulting in enhanced RS drive on the ipsilesional side that might underlie motor recovery and mirror activity.

## 2 Case presentation

**Figure 1:**
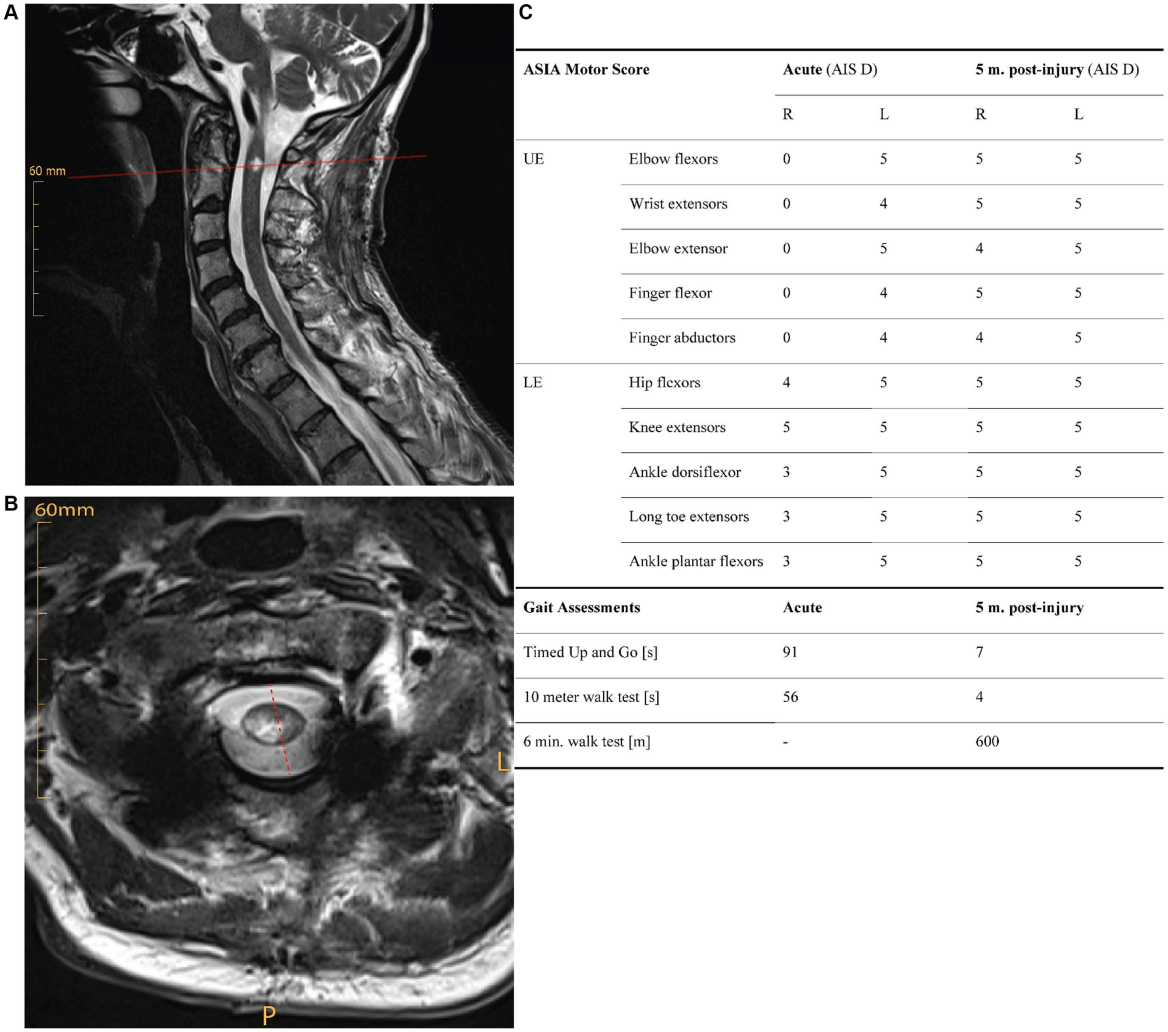
**(A)** Saggital and **(B)** axial T2-weighted magnetic resonance images of the cervical spine acutely (i.e., 35 days) post injury, showing a mainly right-sided spinal lesion. **(C)** Functional recovery over five months post-injury. Clinical measures of muscle strength (ISNCSCI motor score), and walking function (timed-up and go test, 10-meter walk test, 6-min walk test) show substantial initial impairments that are pronounced on the ipsilesional side. All functional tests demonstrate a strong functional recovery over time. AIS, AISA impairment scale; L, left; LE, lower extremities; m., months; P, posterior; UE, upper extremities.

The subject is a 58-year-old male, who presented with an incomplete tetraplegia (ASIA impairment scale (AIS) D) due to a right-sided SCI at neurological level C1, after a bicycle accident. T2-weighted magnetic resonance imaging (MRI performed at day 35 post-injury) showed a high-cervical spinal lesion primarily affecting the right hemicord (Figures 1A,B). Sensory function as measured by the International standards for neurological classification of SCI (ISNCSCI) assessment score showed that mechano-sensation (light touch) was reduced on both sides, indicating partial spinal damage of the contralesional dorsal column. Pain sensation (pinprick) was reduced on the ipsilesional side, and nearly absent contralesionally. The subject showed motor symptoms reflecting a BSS with ipsilesional paresis being more pronounced in the upper compared to the lower extremities (Figure 1C). The patient’s syndrome conforms to BSPS resulting from a mainly unilateral spinal lesion.

The subject showed a substantial degree of functional recovery: He regained the ability to walk independently seven weeks post-injury. Five months after injury, most key muscles of the upper and lower extremities showed full strength according to the ISNCSCI motor examination (Figure 1C). The subject was discharged from inpatient rehabilitation five months post-injury with some persisting motor impairments in the upper ipsilesional extremity.

### 2.1 Functional assessments

Clinical data were assessed during the acute phase (one-month post-injury) as well as five months post-injury, thus allowing to monitor functional recovery over time. The ISNCSCI motor score was used to assess the strength of upper and lower extremity muscles. The ISNCSCI motor score rates muscle strength on a scale from 0 (no strength) to 5 (full strength). Walking function was assessed by standardized clinical tests including the Timed Up and Go test, the 10-meter walk test, and 6-min walk test.

### 2.2 Neurophysiological assessments

RS drive to the ipsi- and contralesional cord was assessed by the StartReact paradigm at five months post-injury. The subject sat in a chair placed 0.3 m in front of a speaker box (ElectroVoice, ELX200, United States). Sound intensity was adjusted with a high-precision sound level meter (Cirrus research, CR162B). First, the subject was presented with five LAS (120 dB, 50 ms, 1,000 Hz) to get familiarized with the loud startling tones. This was followed by a paradigm of 30 stimuli with a randomized order of 19–21 MAS (82 dB, 50 ms, 1,000 Hz) and 9–11 LAS. Varying numbers of LAS and MAS were used to prevent anticipation of the imperative stimulus towards the end of the blocks. Each stimulus was preceded by a warning stimulus (92 dB, 50 ms, 500 Hz) with varying time intervals between the warning stimulus and LAS or MAS (1500–3,000 ms). Acoustic stimuli were generated using a custom-made Simulink application (Matlab R2021b, Mathworks Inc., Natick, United States). The subject was instructed to perform different movements as fast as possible upon LAS or MAS. There was no instruction on the extent of movement. In total, the patient performed six experimental StartReact blocks [elbow flexion (left & right), elbow extension (left & right), and ankle dorsiflexion (left & right)]. Surface EMG signals were recorded bilaterally from the sternocleidomastoid, biceps brachii, triceps brachii, and tibialis anterior muscles using bipolar Ag-AgCI surface EMG Electrodes (H124SG, Kendall). The EMG signal was sampled at 2000 Hz and recorded using a wireless EMG system (Myon Aktos, Cometa Systems, Bareggio, Italy). Acutely and six months post-injury, a clinical neurophysiological assessment was performed. Transcranial magnetic stimulation (single-pulse TMS at 100% stimulator output with double cone coil, Magstim BiStim^2^, The Magstim Co Ltd., Whitland, UK) was performed one and six months post-injury in the framework of clinical routine assessments, and therefore deviates from classical research protocols. MEPs were assessed to probe CS drive to biceps brachii and tibialis anterior muscles on the ipsi- and contralesional side. To evoke MEPs in the biceps brachii, the coil was placed at the vertex, and 4 cm laterally to the left (for ipsi-) or right (for contralesional biceps). For MEPs in the tibialis anterior, the coil was placed at the vertex. MEPs were applied without muscle pre-contraction. Per muscle and time point, two to six stimulations were applied.

### 2.3 Data analysis

EMG muscle onset was defined as EMG activity surpassing baseline EMG activity ± two standard deviations (SD). Baseline EMG activity was measured 100 ms before stimulus release. Motor reaction time was calculated from the time between stimulus onset and muscle onset. The StartReact effect was calculated by subtracting the median reaction time of LAS trials from the median reaction time of MAS trials. MEPs were bandpass filtered from 10 to 500 Hz and rectified.

Mirror activity was analyzed based on EMG activity during StartReact trials. Specifically, EMG activity was assessed in homologous muscles of the contralateral side during a task on the affected side. The time point of muscle onset in the voluntary muscle was determined (T_onset_). The mean area under the curve (AUC) for 100 ms after T_onset_ was calculated in the EMG signal of the mirror muscle (AUC_mirror_). Background EMG activity was defined as AUC in the time window of 1 s before T_onset_ in the EMG signal of the mirror muscle (AUC_background_). AUC values were time normalized. Mirror activity was expressed as ratio of AUC_mirror_ to AUC_background_ in percent [adapted from Cincotta et al. (25)]. Values above 100% indicate the presence of mirror activity in the muscle contralateral to the voluntary movement.

### 2.4 Statistical analysis

Statistical analysis was performed using R (Version 4.2.3, RStudio, Inc.), with the level of significance set at *p <* 0.05 for all statistical tests. Given the non-normal data distribution of reaction time values (assessed with a Shapiro–Wilk-Test), the Mann–Whitney *U* test was performed to compare reaction times in response to MAS and LAS for each task. StartReact effects were quantified by the Wilcoxon signed-rank test after randomized down-sampling of MAS trials to the number of LAS trials. Cohen’s d effect sizes were calculated.

Mirror activity was compared between LAS and MAS using the Mann–Whitney *U* test. Due to a lack of a significant difference, MAS and LAS trials were combined for the analysis of mirror activity. Mirror activity for the contralesional muscles was analyzed during tasks of the homologous ipsilesional muscles. Mirror activity values were compared using a Kruskal-Wallis test. A pairwise comparison was performed using a Bonferroni-corrected Dunn’s test and Hedges’s g effect sizes were calculated.

## 3 Results

### 3.1 Functional assessments

All clinical tests demonstrated a substantial functional recovery of the subject over five months post SCI (Figure 1C). The ISNCSCI motor score demonstrateda complete paralysis of the upper extremity muscles and a partial paresis of lower extremity muscles on the ipsilesional side acutely post-injury. In contrast, muscle strength on the contralesional side was well preserved. Whereas muscle strength of the ipsilesional elbow flexors and ankle dorsiflexors recovered completely, persisting deficits occurred in the ipsilesional elbow extensor and finger abductor (M. abductor digiti minimi) at five months post-injury. Light touch sensation was reduced bilaterally (ipsi-: 25/56 points; contralesional: 25/56 points) acutely post-injury, and only showed minor recovery over time (ipsi-: 26/56 points; contralesional: 28/56 points). Pain sensation was disproportionally reduced on the contra- (4/56 points) vs. ipsilesional side (36/56 points) acutely, and did not recover over a period of five months (ipsi-: 27/56 points; contralesional: 0/56 points). Clinical gait measures demonstrated obvious walking impairments in the acute phase after injury, which recovered substantially over five months (Figure 1C).

### 3.2 StartReact

**Figure 2:**
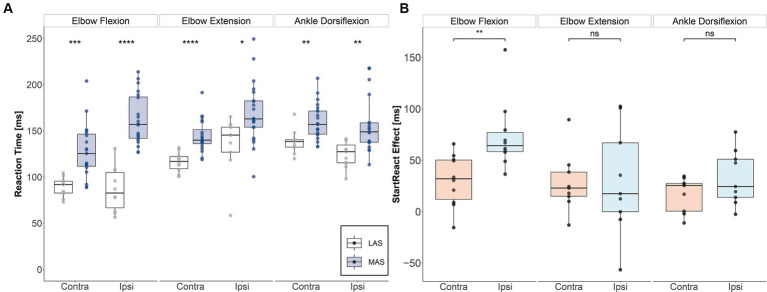
StartReact effects for upper and lower extremity muscles. **(A)** Median reaction times for each task and side for moderate acoustic stimuli (MAS) and loud acoustic stimuli (LAS). (Significance levels of Mann–Whitney *U* test: * *p ≤* 0.05, ** *p ≤* 0.01, *** *p ≤* 0.001, **** *p ≤* 0.0001). **(B)** StartReact effects as measured by the difference in reaction time between MAS and LAS trials for each task and side. There was an enhanced reticulospinal gain in the ipsi- vs. contralesional biceps brachii muscle five months after SCI. Contra, contralesional; Ipsi, ipsilesional; LAS, loud acoustic stimuli; MAS, moderate acoustic stimuli.

The subject demonstrated significant StartReact effects in all assessed muscles on both sides (Figure 2A). Accordingly, reaction times were significantly faster in response to LAS than MAS (biceps brachii left (contralesional): *U* = 17, *p* < 0.001, *d* = 1.61; biceps brachii right (ipsilesional): *U* = 2, *p* < 0.001, *d* = 2.87; triceps brachii left: *U* = 11, *p* < 0.001, *d* = 1.82; triceps brachii right: *U* = 39, *p* = 0.013, *d* = 1.06; tibialis anterior left: *U* = 31.5, *p* = 0.005, *d* = 1.2; tibialis anterior right: *U* = 34, *p* = 0.003, *d* = 1.23; Mann–Whitney *U* tests).

Figure 2B shows that the StartReact effect, which reflects RS drive, was substantially enhanced on the ipsilesional compared to the contralesional side for the biceps brachii (V = 1, *p* = 0.004, *d* = 1.37). In contrast, the StartReact effect did not differ between the ipsi- vs. contralesional side in the triceps brachii (V = 21, *p* = 0.91, *d* = 0.04) nor in the tibialis anterior (V = 8, *p* = 0.098, *d* = 0.58; Wilcoxon signed-rank test). Cortical motor-evoked potentials (MEPs):

### 3.3 Cortical motor-evoked potentials (MEPs)

**Figure 3:**
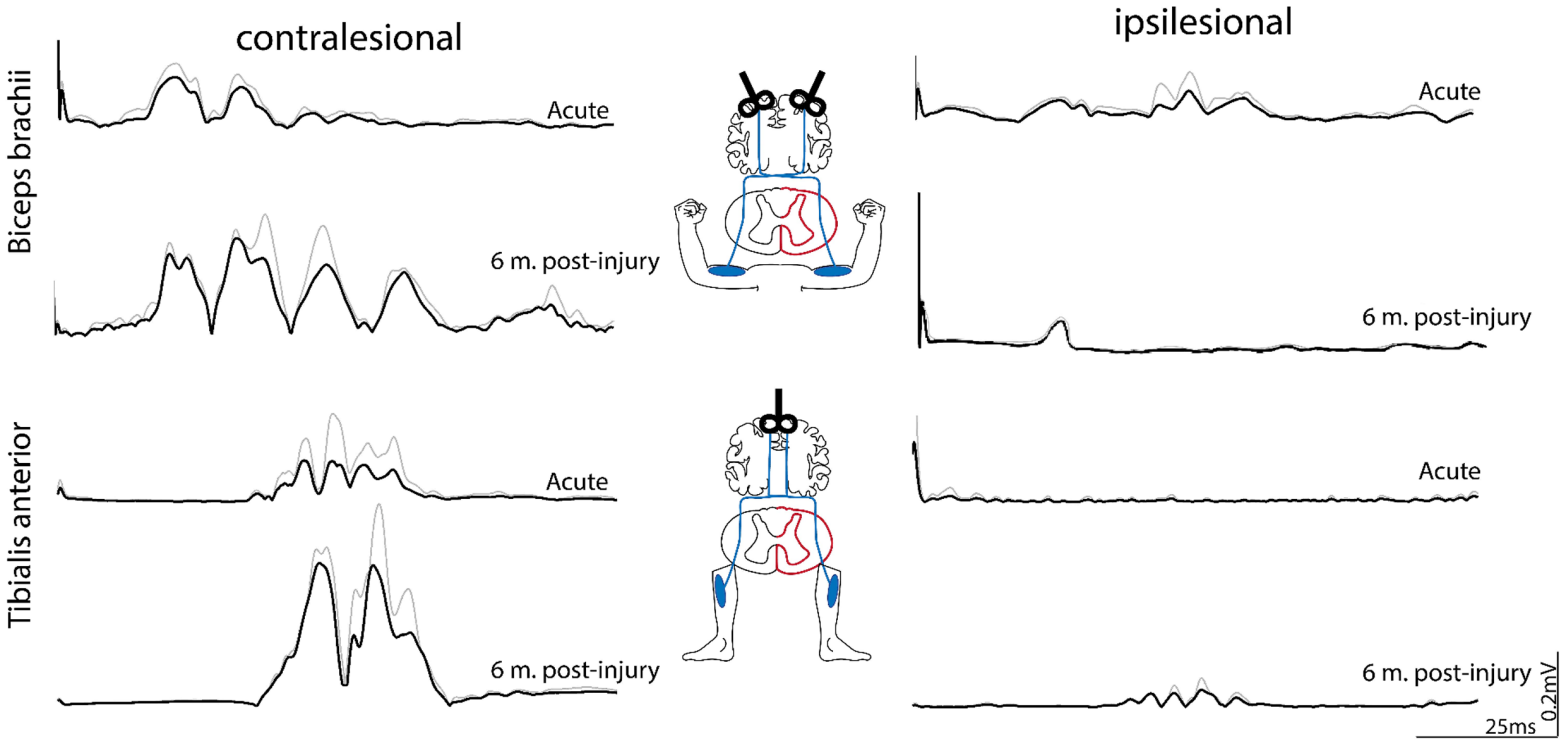
Motor-evoked potentials (MEPs) assessing corticospinal drive to biceps brachii and tibialis anterior muscles. Transcranial magnetic stimulation (TMS) was applied over the relative cortical representation of the arm and leg for both the ipsi- and contralesional side. Acutely after injury (1 month post-injury), MEPs were observed in the contralesional biceps and tibialis anterior. Low-amplitdue MEP occurred in the ipsilesional biceps, whereas no MEPs were observed in the ipsilesional tibialis anterior. Six months post-injury, MEPs were enhanced in the contralesional muscles, whereas the ipsilesional biceps brachii did not reveal enhanced MEPs compared to acute time points. In contrast to acute time points, MEPs re-occurred in the ipsilesional tibialis anterior 6 months post-inury, indicating restoration of CS drive to this muscle. MEP responses represent grand averages (black) + standard deviations (gray) of multiple stimulations (two to six per muscle). m, month(s).

Acutely after injury, MEP latencies were delayed in biceps brachii muscles bilaterally [contralesional: 17.1 ms; ipsilesional: 18.2 ms; cutoff pathological latency: 12 ms (26)] and in the contralesional tibialis anterior [33.6 ms; cutoff pathological latency: 32.5 ms (26)]. No MEPs could be evoked in the ipsilesional tibialis anterior acutely post-injury (Figure 3). MEP latencies after six months post-injury were similar to the acute values in biceps brachii muscles (contralesional: 14.2 ms; ipsilesional: 19.5 ms) and the contralesional tibialis anterior (33.7 ms). Compared to the acute phase, MEP amplitude was unchanged in the ipsilesional biceps (acute: 0.05 mV; 6 months: 0.06 mV), but slightly enhanced on the contralesional side (acute: 0.11 mV; 6 months: 0.19 mV). MEP amplitude in the contralesional tibialis anterior was increased after six months compared to acutely post-injury (acute: 0.1 mV; 6 months: 0.33 mV; Figure 3). Interestingly, MEP responses were observed in the ipsilesional tibialis anterior six months post-injury (latency: 33.8 ms; amplitude: 0.04 mV), indicating partial restoration of CS drive to ipsilesional lumbar motoneuron pool of the tibialis anterior. Motor nerve conduction velocity of the ulnaris and tibialis nerves were not impaired acutely and six months post-injury, implying that there was no impairment of the peripheral nervous system.

### 3.4 Mirror activity

**Figure 4:**
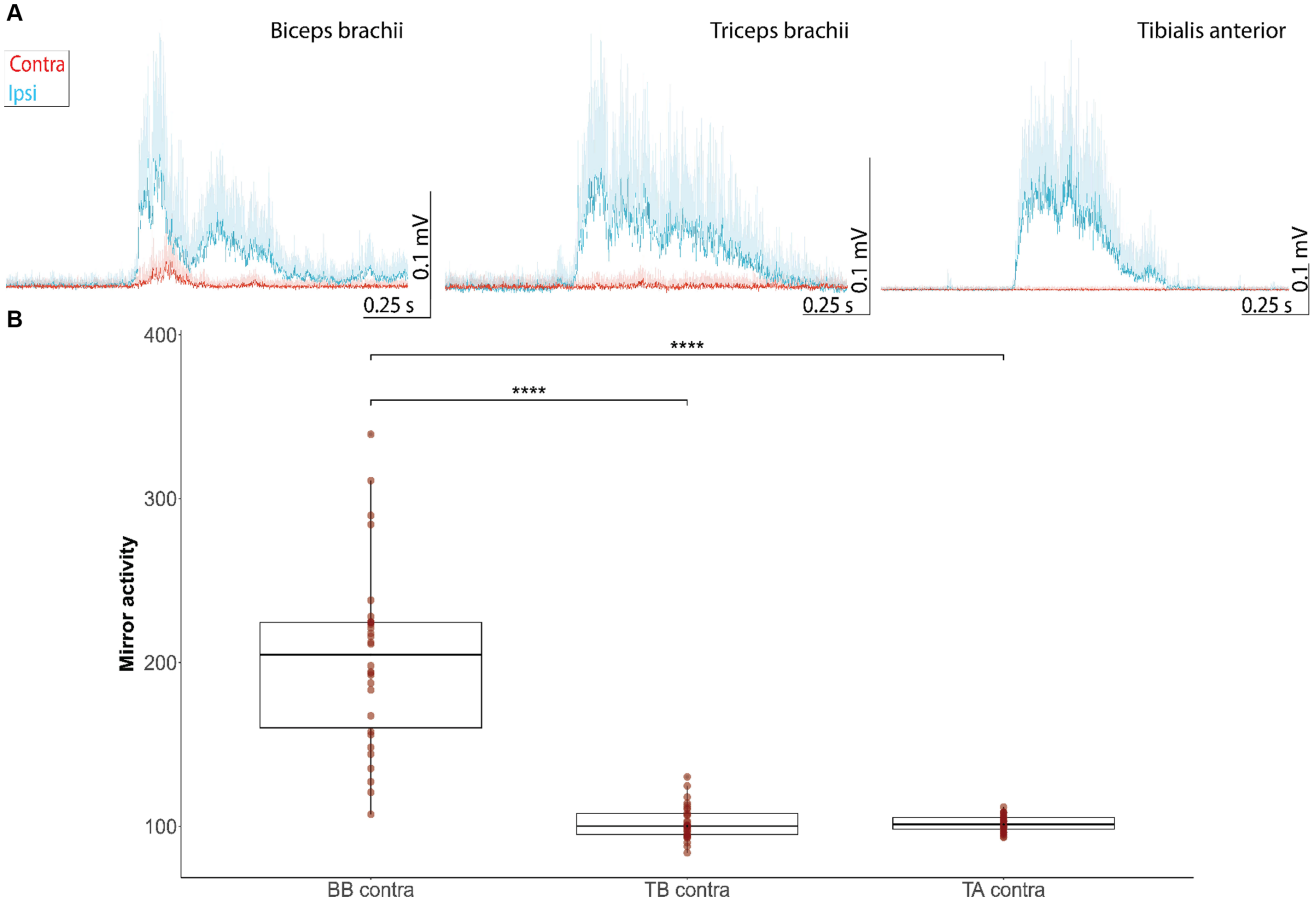
Mirror activity in muscles of the contralesional side evoked by movements of homologous muscles on the more impaired, ipsilesional side. **(A)** EMG responses represent grand averages + standard deviations of the ipsi- (blue line) and contralesional muscles (red curve) during movements of the ipsilesional joints. Mirror activity is observed in the contralesional biceps brachii, but not triceps brachii and tibialis anterior muscle. EMG traces were offset corrected to the baseline EMG signal before muscle onset (−1,000 ms to −1 ms before muscle onset). **(B)** Mean AUC of the contralateral EMG during 100 ms after muscle onset in the voluntary muscle, expressed as a percentage of the mean background AUC level in the contralateral muscle. Values *<*100% indicatemirror activity (significance levels of Dunn-Bonferroni-Test: * *p ≤* 0.05, ** *p ≤* 0.01, *** *p ≤* 0.001, **** *p ≤* 0.0001). AUC, Area under the curve; BB, biceps brachii; contra, contralesional; TA, tibialis anterior; TB, triceps brachii.

Mirror acitivity was examined on the contralesional side during the voluntary contraction of the homologous muscles on the right ipsilesional side (Figure 4). Mirror activity was observed only in the biceps brachii muscles, but not the triceps brachii and tibialis anterior (Figure 4A). The amount of mirror activity was different across muscles (H (3) = 65.7, *p* < 0.001, *η*2 = 0.5; Kruksal-Wallis Test), with biceps brachii showing higher values than the other muscles (biceps left vs. triceps left: *z* = *−*7.0 *p* < 0.001, Hedges’s g = 2.5; biceps left vs. tibialis anterior left: *z* = *−*6.9, *p* < 0.001, Hedges’s g = 2.55; Mann–Whitney *U* Test; Figure 4B).

## 4 Discussion

The subject presented with a BSPS, with severe initial motor impairment on the ipsilesional side. The subject showed substantial motor recovery over five months post-injury. Superior recovery of biceps brachii vs. triceps brachii function agrees with earlier reports (27). Recent findings by Sangari and Perez (13) provide evidence that increased RS drive to the biceps brachii, but not the triceps brachii might be the underlying mechanism for the better recovery of elbow flexion vs. extension in subjects with cervical incomplete SCI. Indeed, preclinical and clinical findings point towards a strong RS drive to elbow flexors (13, 17, 20, 28). Our data provide supportive evidence for the role of RS plasticity in the functional recovery of ipsilesional biceps brachii function by showing enhanced RS drive to the ipsi- vs. contralesional biceps brachii. The fact that MEPs in the ipsilesional biceps brachii did not normalize over time suggests that recovery of elbow flexion is not caused by CS plasticity, supporting earlier reports (29). Interestingly, MEP normalization was observed in the ipsilesional tibialis anterior, which is in line to previous findings in patients with BSS (30). In contrast, only moderate left-to-right difference in the StartReact effects was present in this muscle. The novel findings from this participant with BSPS suggest that recovery of ipsilesional ankle dorsiflexion is predominantly driven by CS plasticity. This is in line with reports demonstrating that the tibialis anterior is under strong CS control (31, 32) and that CS plasticity promotes recovery of ankle dorsiflexion (33). Our findings indicate that the neuroplastic mechanisms underlying motor recovery may differ between various muscles, and that the neuroplastic potential of the RS system might be pronounced in proximal flexor muscles (34). Combinatory neurophysiological assessments including StartReact and MEPs will be required to further disentangle the role of neuroplasticity in descending motor systems for functional recovery after CNS injury.

Our findings support the idea of meaningful contributions of the RS system to functional recovery in incomplete SCI reported in preclinical (8–10) and clinical studies (12, 13). While the StartReact paradigm does not allow to differentiate between compensatory or regenerative plasticity, our data imply that RS drive is enhanced for particular muscles on the largely denervated ipsilesional side and that this neuroplastic adaption is associated with functional recovery in individuals with SCI. The presence of a notable StartReact effect on the ipsilesional side five months after a half-sided spinal lesion might be explained by the high proportion of bilateral RS projections to the spinal cord, including numerous midline-crossing projections both above and below the lesion (9). This diffuse projection pattern allows the RS system to convey motor drive to the ipsilesional spinal cord below the injury. Additionally, enhanced StartReact effects in the ipsi- vs. contralesional biceps brachii suggests neuroplastic adaptions in the RS system over time. However, as there is no available data on the StartReact effect acutely post-injury, the underlying mechanisms of augmented RS drive remain unknown. MEPs have been examined in the framework of routine clinical assessments and, therefore, have not been performed for triceps brachii. We are, therefore, not able to discuss the role of CS plasticity on the functional recovery of this muscle.

Beneficial effects of RS plasticity on motor recovery partially contrast findings in stroke patients where RS plasticity is sometimes associated with maladaptive phenomena such as associated movements or spasticity (35–37). This negative association, however, might also be driven by the fact that both RS plasticity and maladaptive features are related to the severity of corticospinal tract damage (38). The discrepant effects of RS plasticity in stroke and SCI might be explained by the deviating extent of loss in cortical motor control: Patients with stroke often show hyperexcitability of the reticular formation which is triggered by cortico-reticular disinhibition (39). In contrast, regulation of the reticular formation by cortical structures is preserved in patients with SCI, which might allow the RS system to contribute to meaningful recovery in SCI patients (12, 29).

Increased RS drive to biceps brachii motoneurons in the ipsilesional hemicord was accompanied by the occurrence of mirror activity in the contralesional biceps. Mirror activity has previously been linked to an upregulated RS drive in response to CS tract damage (36). Ejaz et al. (23) demonstrated that the occurrence of mirror activity in the biceps provides supporting evidence for an enhanced bilateral RS drive to spinal motoneurons. Interestingly, mirror activity only occurred in the muscle revealing the highest StartReact effect, which further supports that mirror activity is, at least in part, mediated by the RS system.

There are some limitations regarding this report. In contrast to the clinical routine assessments (such as gait assessments, TMS etc.), StartReact measurements have not been performed acutely post-injury, because our research team was not aware of this patient case at this stage. However, considering the complete paralysis of ipsilesional biceps and triceps brachii muscles acutely after injury, RS drive to these muscles (as measured by the StartReact paradigm) can likely be assumed to be absent at this time. Another limitation concerns the accuracy of the unilateral spinal lesion. Although sensory assessments and MEPs indicate that the syndrome does not conform to a pure BSS, the canonical motor features of the BSS, which are of main interest for this report, are present in a form which is rare. Despite signs of weak contralesional CS tract damage, CS impairment seems clearly more pronounced on the ipsi- than contralesional side as indicated by strongly reduced or absent MEP amplitudes in the ipsilesional biceps brachii and tibialis anterior acutely post-injury. This is in line with neuroimaging data indicating a high-cervical, primarily right-sided spinal lesion. Therefore, the functional, electrophysiological, and neuroimaging findings suggest an asymmetric, largely one-sided spinal lesion that mimics several sensorimotor features of the BSS.

## 5 Conclusion

The findings of this case report suggest that RS plasticity occurring after incomplete SCI can be assessed by the StartReact paradigm. The BSPS provides a unique opportunity to compare neuroplastic adaptations in the RS system between the largely spared (contralesional) and impaired (ipsilesional) side. The data imply that enhanced RS drive is mainly found in the ipsilesional biceps brachii where it is related to motor recovery and the occurrence of mirror activity. Further research is needed to gain more insights into the contribution of the RS and CS system to functional recovery and to disentangle the beneficial and maladaptive effects of RS plasticity in human SCI and other neurological conditions.

## Data availability statement

The original contributions presented in the study are included in the article/supplementary material, further inquiries can be directed to the corresponding author.

## Ethics statement

The study was approved by the Ethics Committee of the Canton Zurich. The study was conducted in accordance with the guidelines of the Declaration of Helsinki and Good Clinical Practice. Written informed consent was obtained from the individual for the publication of any data included in this article.

## Author contributions

AE: Data curation, Formal analysis, Investigation, Methodology, Visualization, Writing – original draft, Writing – review & editing. MR: Investigation, Writing – review & editing. BZ: Investigation, Writing – review & editing. AC: Investigation, Writing – review & editing. LF: Conceptualization, Formal analysis, Funding acquisition, Investigation, Methodology, Supervision, Writing – original draft, Writing – review & editing.
